# Supplementary material for: Characterization and Antioxidant Activity of Exopolysaccharides Produced by Lysobacter soyae sp. nov Isolated from the Root of Glycine max L
Source: Microorganisms. 2023 Jul 27;11(8):1900. doi: 10.3390/microorganisms11081900 (PMC10456730; doi:10.3390/microorganisms11081900)
Supplement: Supplementary file 1 [file microorganisms-11-01900-s001.zip › microorganisms-2506990-supplementary.pdf]

Characterization and antioxidant activity of exopolysaccharides produced by *Lysobacter soyae* sp. nov isolated from the root of *Glycine max* L.

Inhyup Kim, Geeta Chhetri, Yoonseop So, Sunho Park, Yonghee Jung, Haejin Woo, and Taegun Seo *

Department of Life of Science, Dongguk University-Seoul 10326, Republic of Korea

*Correspondence: tseo@dongguk.edu; +82-31-961-5135

Figure S1. Polar lipid profiles of strain CJ11^{T} obtained through two-dimensional thin-layer chromatography. Polar lipids detected by spraying with phosphomolybdic acid. PE, phosphatidylethanolamine; DPG, diphosphatidylglycerol; PG, phosphatidylglycerol; PL, phospholipid. Chloroform–methanol–water (65:25:4; *v*/*v*) was used in the first direction, and chloroform–acetic acid–methanol–water (80:15:12:4; *v*/*v*) was used in the second direction.

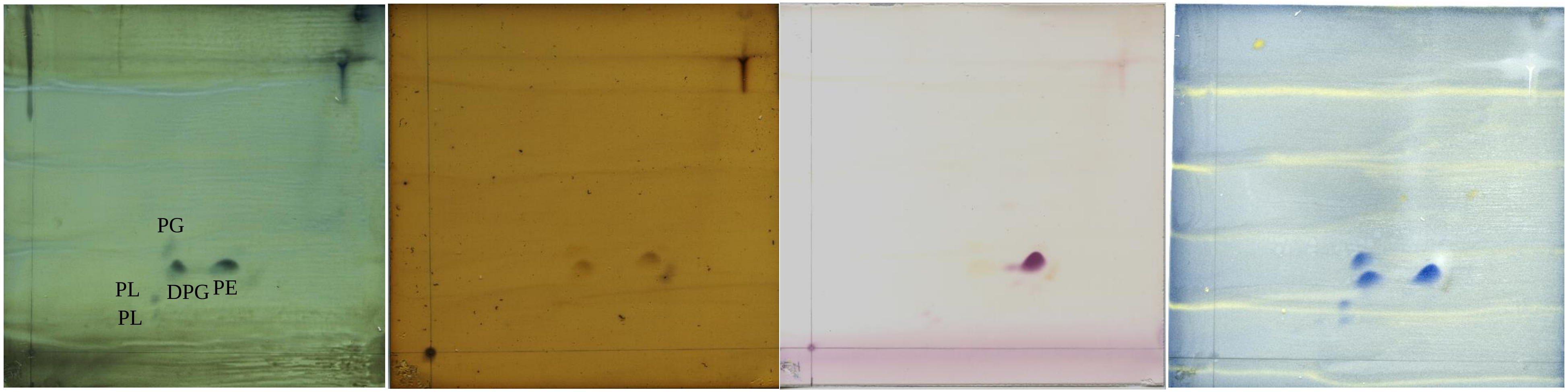

Figure S2. Circular genome map of strain CJ11ᵀ. rRNA genes (light green), tRNA genes (red), tmRNA (sky blue), GC content (yellow), and GC skew (green and purple).

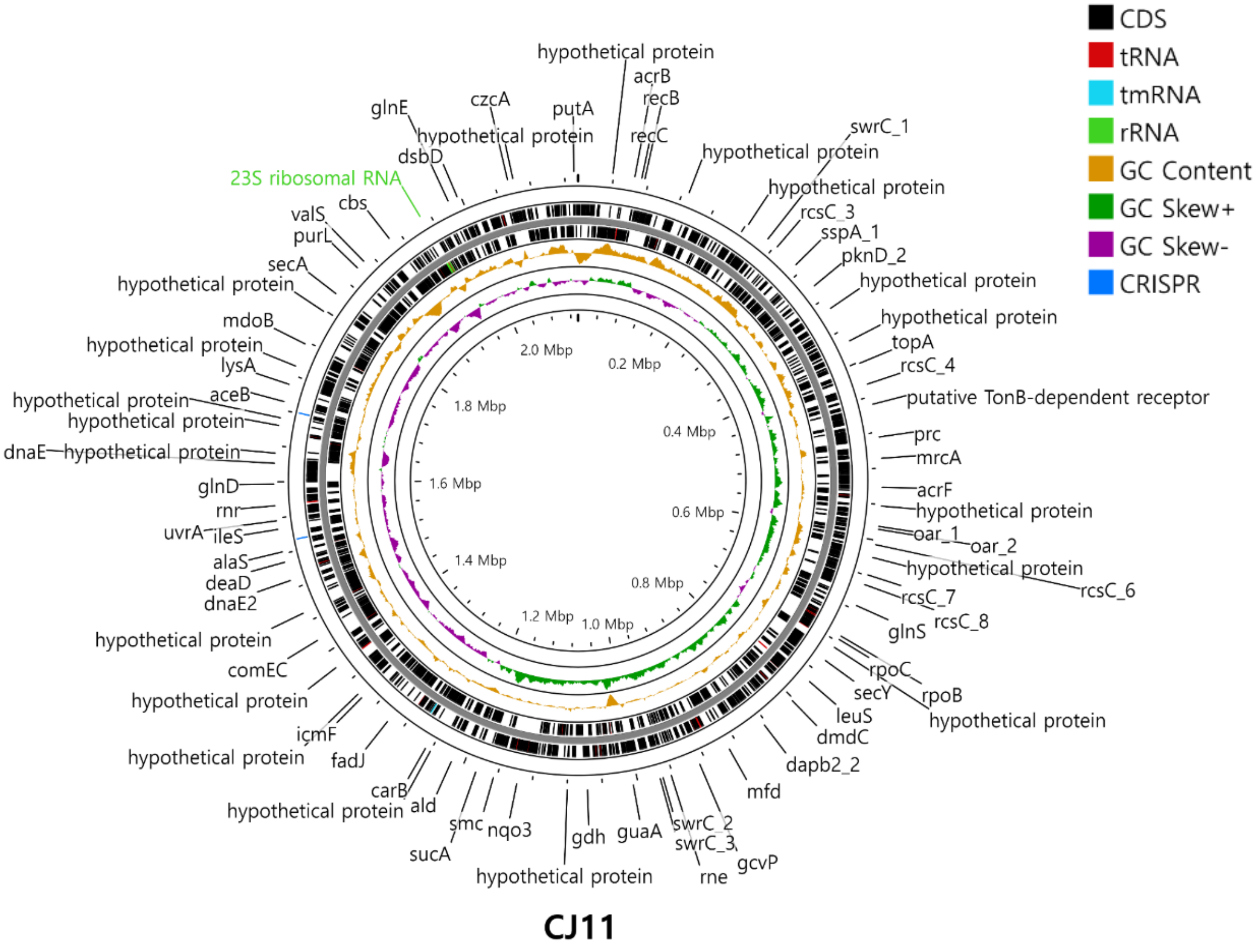

Figure S3. Phylogenomic tree reconstructed using the coding sequences of 92 protein clusters showing the position of strain CJ11ᵀ. All genome sequences of the 24 related strains are available on the NCBI GenBank and EzBioCloud Whole-Genome databases. Percentage bootstrap values (>80%) are given at the branching points. Genome accession numbers are indicated in parentheses. Bar, 0.05 changes per position.

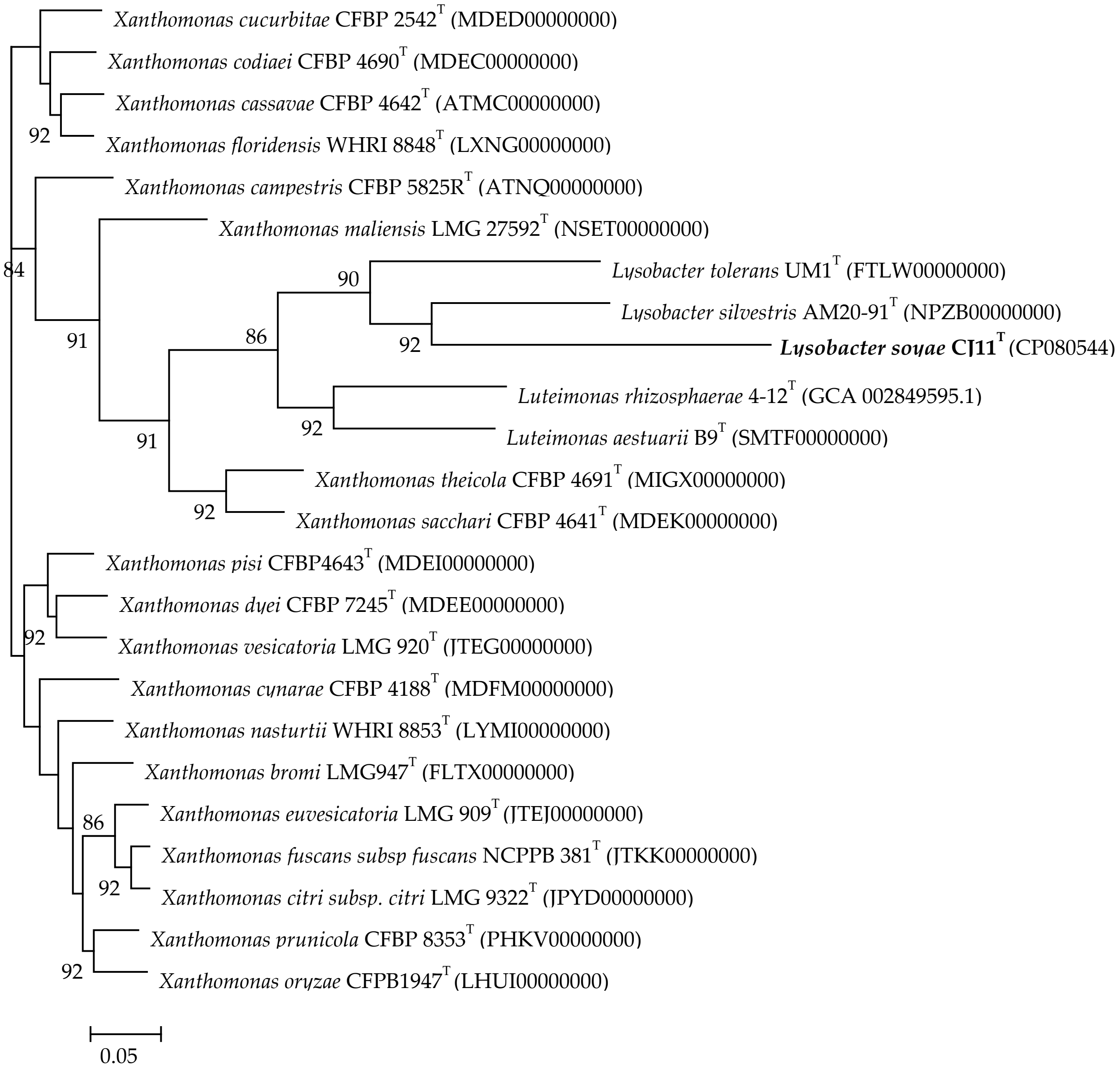

Figure S4. The clusters of orthologous groups (COG) functional category of strain CJ11^T and the phylogenetically related *Lysobacter* species (*L. tolerans* UM1^T and *L. silvestris* AM20-91^T). 1, Strain CJ11^T; 2, *L. tolerans* UM1^T; 3, *L. silvestris* AM20-91^T.

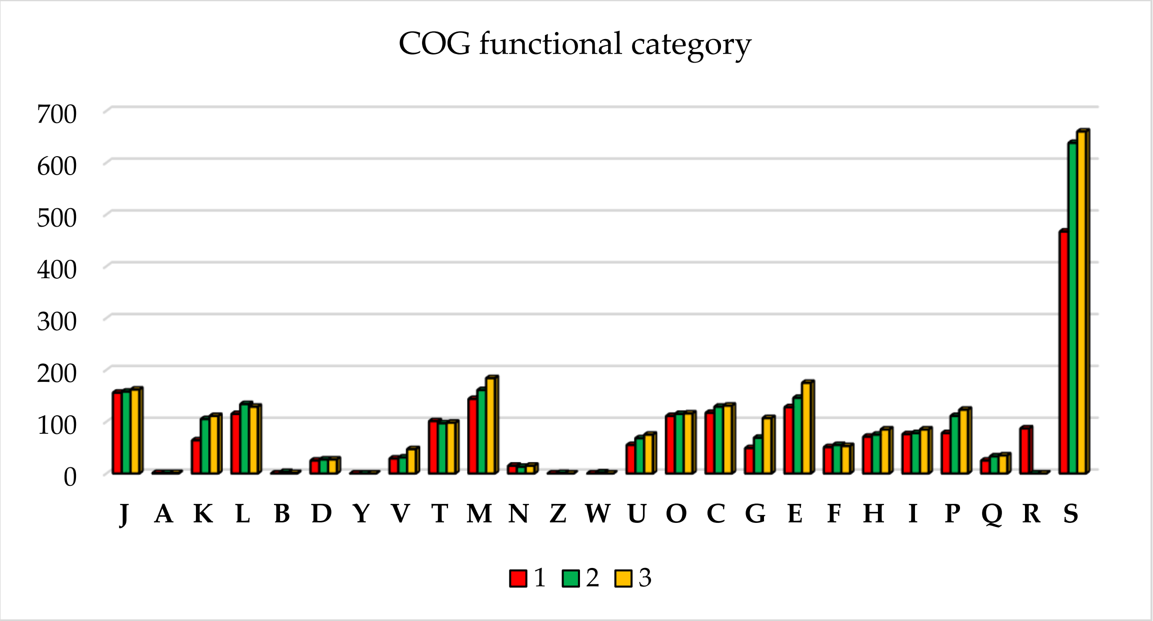

J, translation, ribosomal structure, and biogenesis; **A**, RNA processing and modification; **K**, transcription; **L**, replication, recombination, and repair; **B**, chromatin structure and dynamics; **D**, cell-cycle control, cell division, chromosome partitioning; **Y**, nuclear structure; **V**, defense mechanisms; **T**, signal transduction mechanisms; **M**, cell wall, membrane, envelope biogenesis; **N**, cell motility; **Z**, cytoskeleton; **W**, extracellular structures; **U**, intracellular trafficking, secretion, and vesicular transport; **O**, posttranslational modification, protein turnover, chaperones; **C**, energy production and conversion; **G**, carbohydrate transport and metabolism; **E**, amino-acid transport and metabolism; **F**, nucleotide transport and metabolism; **H**, coenzyme transport and metabolism; **I**, lipid transport and metabolism; **P**, inorganic ion transport and metabolism; **Q**, secondary metabolites biosynthesis, transport and catabolism; **R**, general function prediction only; **S**, function unknown.

Figure S5. The Distilled and Refined Annotation of Metabolism (DRAM) annotation of metagenome-assembled genomes (MAGs) of the phylogenetically related species to the *Lysobacter* species isolated from rhizosphere soils of soybean fields. The color of the heatmap indicates the presence (green) or absence of an associated metabolic function in the genome. 1, strain CJ11^{T}; 2, *L. tolerans* UM1^{T}; 3 *L. silvestris* AM20-91^{T}.

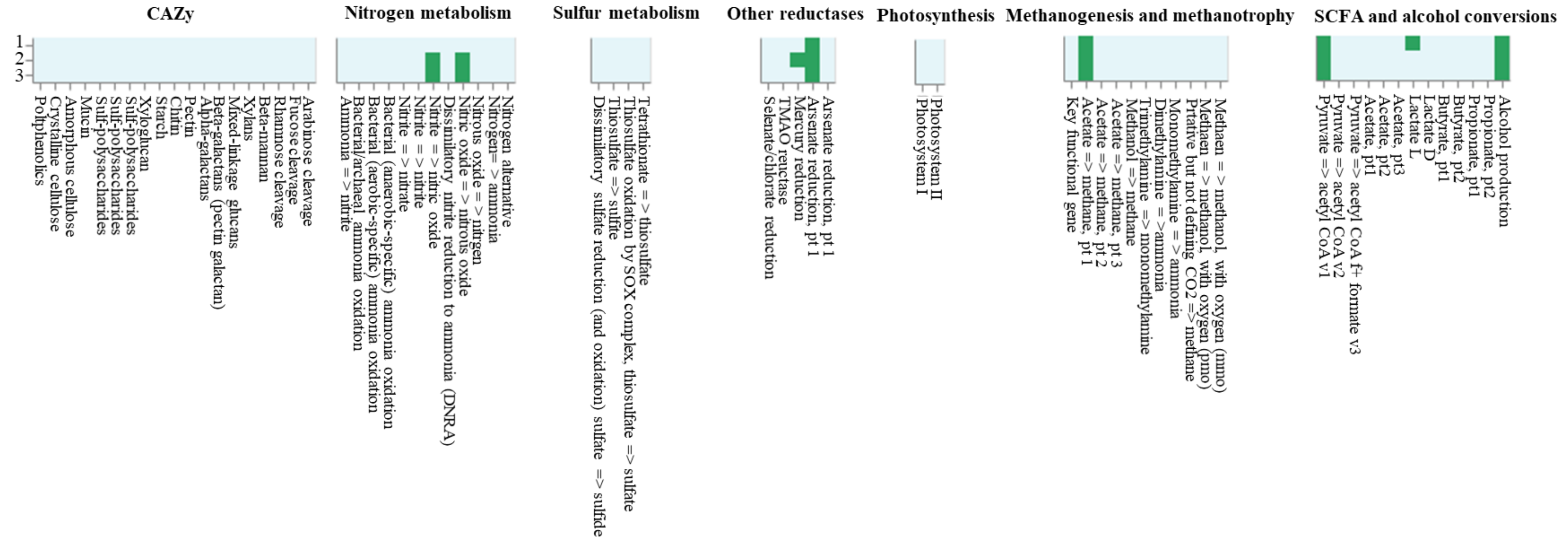

Figure S6. Average Mw distributions of EPS.

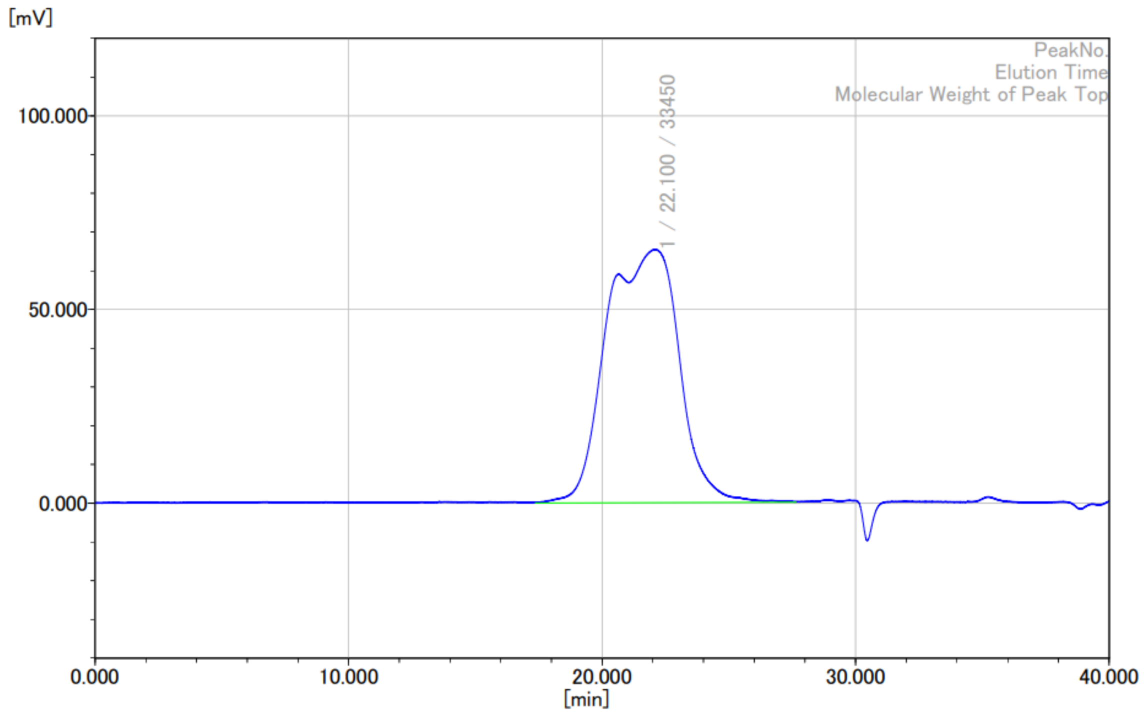

Figure S7. EPS produced by *Lysobacter* sp. CJ11^{T} was evaluated for monosaccharide composition using bio-liquid chromatography (Bio-LC). Retention time is indicated above each peak. (a) EPS; (b) monosaccharide standards.

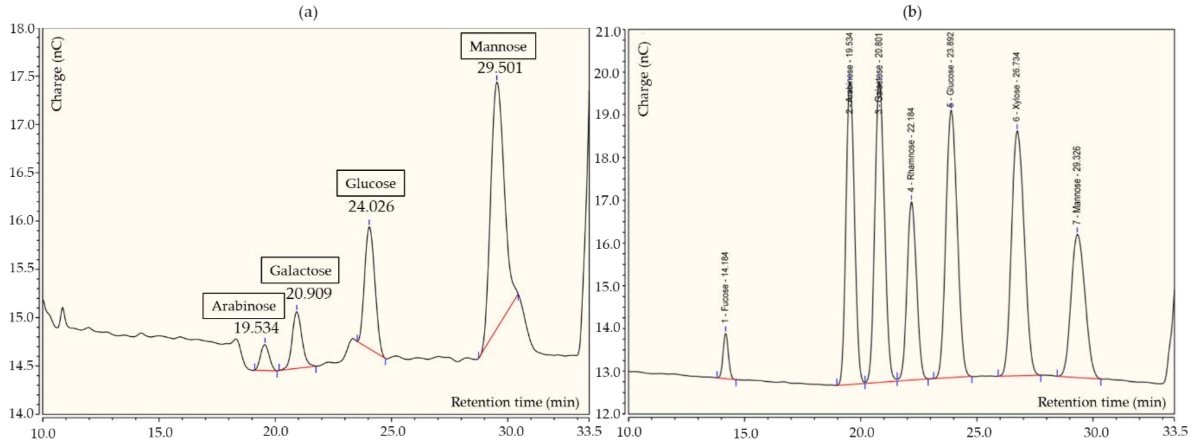

Table S1. Differentiating characteristics of strain CJ11^T and other *Lysobacter* species: 1, CJ11^T; 2, *Lysobacter tolerans* UM1^T; 3, *Lysobacter silvestris* AM20-91^T. All dates are from this study. On the API ZYM strips, all strains were positive for alkaline phosphatase, leucine arylamidase, esterase lipase (C8), acid phosphatase, and naphthol-AS-BI-phosphohydrolase; however, they were negative for *α*-chymotrypsin, *β*-galactosidase, *β*-glucuronidase, *α*-fucosidase, and esterase (C4). On the API 20NE strips, all strains were negative for nitrate reduction, indole production, fermentation of D-glucose, and L-arginine. Although assimilation of D-glucose, L-arabinose, D-mannitol, caprate, and phenylacetate were negative for all strains, they were all positive for *β*-galactosidase activity. No strain could hydrolyze esculin, gelatin, starch, DNase, Tween-80, and CM-cellulose. +, positive; –, negative; w+, weakly positive.
* From whole-genome sequencing (WGS) for genomic sequences.

Characteristics	1	2	3
Ranges for growth			
NaCl (direction *w*/*v*, %)	0–2	0–1	0–1
pH (optimal)	5–11 (7–8)	5–10 (7)	2–12 (8)
Temperature (°C)	15–37	25–37	10–30
Grown media	TSA, NA, LB, R2A	NA, R2A	TSA, NA, LB, R2A
Hydrolysis of			
Tween-20	+	–	–
Casein	–	w+	+
Enzyme activities:			
Lipase (C14)	–	+	–
Valine arylamidase	–	+	+
Trypsin	–	+	+
α-galactosidase	–	+	+
α-glucosidase	–	+	+
β-glucosidase	–	–	+
N-acetyl-*β*-glucosaminidase	–	+	+
α-mannosidase	–	–	+
Assimilation of:			
N-acetyl-glucosamine	–	–	+
D-maltose	–	+	+
Potassium gluconate	+	–	–
Adipate	+	–	–
Malate	+	–	–
Trisodium citrate	+	–	–
DNA G + C content (mol.%)	59.2*	61.6*	63.4*

Table S2. Genome sequence features of strain CJ11^T and other *Lysobacter* species. Data for reference strains were retrieved from NCBI. 1, *Lysobacter donggukensis* CJ11^T; 2, *Lysobacter tolerans* UM1^T; 3, *Lysobacter silvestris* AM20-91^T.

	Accession No.	Size (Mbp)	Total genes	Number of coding sequences	G + C content (mol.%)
1. *Lysobacter donggukensis* CJ11^T	CP080544	2.14	2069	2007	59.2
2. *Lysobacter tolerans* UM1^T	FTLW00000000	2.54	2468	2389	61.6
3. *Lysobacter silvestris* AM20-91^T	NPZB00000000	2.85	2638	2566	63.4

Table S3. Average nucleotide identity (ANI) indexes for the genome of the type strain of this species belonging to the family *Lysobacteraceae*. *L*, *Lysobacter*; *X*, *Xanthomonas*; *L*, *Luteimonas*

	**1**	**2**	**3**	**4**	**5**	**6**	**7**	**8**	**9**	**10**	**11**	**12**	**13**	**14**	**15**	**16**	**17**	**18**	**19**	**20**	**21**	**22**	**23**
**1**. *L. donggukensis* CJ11^T																							
**2**. *L. tolerans* UM1^T	71.6																						
**3**. *L. silvestris* AM20-91^T	72.0	74.0																					
**4**. *X. sacchari* CFBP 4641^T	70.7	73.1	73.7																				
**5**. *L.aestuarii* B9^T	70.7	73.4	74.4	76.2																			
**6**. *X. codiaei* CFBP 4690^T	70.8	72.6	73.2	79.9	74.9																		
**7**. *X.campestris* CFBP 5825R^T	70.7	72.5	73.1	79.4	74.5	85.5																	
**8**. *X. cassavae* CFBP 4642^T	70.8	72.5	73.2	79.7	74.8	91.7	85.6																
**9**. *X. cynarae* CFBP 4188^T	70.4	72.2	72.9	79.2	74.5	86.2	85.8	86.5															
**10**. *X. floridensis* WHRI 8848^T	70.6	72.5	73.0	79.6	74.6	91.6	85.5	93.2	86.3														
**11**. *L. rhizosphaerae* 4-12^T	70.9	72.9	74.0	76.4	77.3	74.9	74.4	74.7	74.3	74.4													
**12**. *X. cucurbitae* CFBP 2542^T	70.8	72.5	72.8	79.6	74.9	89.6	85.2	89.5	85.5	89.6	74.6												
**13**. *X. maliensis* LMG 27592^T	70.9	72.3	72.9	79.6	74.5	83.0	82.7	82.9	82.5	83.0	74.5	82.8											
**14**. *X. vesicatoria* LMG 920^T	70.3	72.2	73.0	79.0	74.3	87.2	85.0	87.6	86.2	87.9	74.3	86.6	82.4										
**15**. *X. pisi* CFBP4643^T	70.3	72.3	72.9	79.3	74.8	87.4	85.5	87.6	86.8	89.0	74.7	87.0	82.6	90.3									
**16**. *X. euvesicatoria* LMG 909^T	70.4	70.4	72.9	79.2	74.4	86.4	85.1	86.9	86.4	86.5	74.5	85.8	82.9	86.1	86.5								
**17**. *X. fuscans subsp fuscans* NCPPB 381^T	70.6	72.5	73.0	79.2	74.4	86.3	84.9	86.6	86.5	86.4	74.3	85.7	82.5	86.1	86.6	93.9							
**18**. *X. nasturtii* WHRI 8853^T	70.7	72.6	73.0	79.4	74.7	86.9	85.5	87.2	87.5	87.2	74.3	86.2	82.7	87.4	87.4	88.7	88.8						
**19**. *X. prunicola* CFBP 8353^T	70.2	72.1	72.8	78.9	74.1	86.1	84.9	86.4	86.4	86.4	74.1	85.6	82.3	86.2	87.6	89.8	89.7	88.7					
**20**. *X. citri subsp. citri* LMG 9322^T	70.8	72.3	72.9	79.2	74.3	86.2	84.8	86.7	86.4	86.4	74.4	85.8	82.6	86.0	86.5	94.0	96.1	88.8	89.9				
**21**. *X. theicola* CFBP 4691^T	70.5	73.2	73.8	86.9	76.3	80.1	79.6	79.7	79.5	79.6	76.5	79.7	79.5	79.3	79.6	79.4	79.5	79.4	79.1	79.2			
**22**. *X. bromi* LMG947^T	70.4	72.4	72.6	78.9	74.2	86.5	85.1	86.8	86.6	86.9	74.3	85.9	82.4	86.8	86.9	89.3	89.2	88.9	89.6	89.3	79.2		
**23**. *X. dyei* CFBP 7245^T	70.4	72.0	72.9	79.2	74.3	87.2	85.5	87.4	86.6	88.1	88.1	86.5	82.4	90.6	91.4	86.2	86.2	87.4	86.5	86.3	79.5	86.8	
**24**. *X. oryzae* CFPB1947^T	70.6	71.8	72.7	78.9	74.4	86.2	85.0	86.3	86.4	86.3	74.1	85.4	82.7	86.0	86.4	90.2	89.9	88.8	91.2	89.9	79.2	89.5	86.2
